# Exerting Explanatory Accounts of Safety Behavior of Older Construction Workers within the Theory of Planned Behavior

**DOI:** 10.3390/ijerph16183342

**Published:** 2019-09-10

**Authors:** Lu Peng, Alan H.S. Chan

**Affiliations:** Department of Systems Engineering and Engineering Management, City University of Hong Kong, Kowloon 999077, Hong Kong, China; alan.chan@cityu.edu.hk

**Keywords:** older construction worker, safety behavior, organizational and personal factors, theory of planned behavior

## Abstract

Older construction workers are vulnerable to accident risks at work. Work behavior affects the occurrence of accidents at construction sites. This study aims to investigate the organizational and personal factors that underlie the safety behaviors of older construction workers considering their age-related characteristics. A cross-sectional questionnaire survey, which involves 260 older construction workers (aged 50 and over), was conducted, and an integrative old-construction-worker safety behavior model (OSBM) was established on the basis of the theory of planned behavior (TPB). Results showed that the OSBM provides a considerably good explanation of the safety behaviors of older construction workers. The explained variances for safety participation and compliance are 74.2% and 63.1%, respectively. Subjective norms and perceived behavioral control are two critical psychological drivers that proximally affect the safety behaviors of workers. Moreover, safety knowledge, management commitment, and aging expectation are the distal antecedents that significantly influence psychological drivers. This study proves the mediating role of psychological factors on predicting safety behaviors among older construction workers, thereby promoting an understanding of “how” and “why” their safety behaviors occur. Furthermore, the identified effects of several critical organizational and personal factors, particularly age-related factors, provide new insights into the safety behaviors of older construction workers.

## 1. Introduction

Workforce aging has become a serious global issue. A high percentage of older workers and a considerable labor shortage in Hong Kong have challenged the local construction industry. A survey of the Hong Kong Construction Association reported that over 43% of local construction workers are at least 50 years old, while the average shortage rate of construction labor in 2013 and 2017 was 11% [1]. Construction is one of the physically demanding industries. Construction workers face major occupational hazards daily. In 2018, 87.5% of fatal industrial accidents in Hong Kong occurred in the construction industry, while accidents at construction sites accounted for 33.4% of all industrial accidents [2]. Past research has suggested that older workers suffer from higher occupational safety risks than their young counterparts [3]. An analysis of occupational accidents involving construction scaffolding by Sawicki and Szóstak indicated that the most common age group of injured people are those aged 46–50 years [4]. Moreover, older construction workers face more risk to occupational accidents than older workers in other business sectors [3].

Fleming and Lardner [5] and Shin et al. [6] stated that most occupational accidents are caused by work behavior. The Commissioner for Labor of Hong Kong has found that work behavior is the root cause of accidents at construction sites [7]. However, evidence exists that many construction workers do not follow safety regulations to take personal protective measures [8,9]. Preventive interventions are essential to identify the factors that contribute to the promotion of safety behaviors [10]. Although some studies have sought to identify the related factors (e.g., impact of supervisor, management commitment, and safety knowledge) on safety behaviors for general [11,12,13] and temporary [14] construction workers, and the factors influencing risk-taking behaviors of general construction workers [15,16], no study has specifically examined the factors or mechanisms underlying the safety behaviors among older construction workers. Bohle et al. indicated that age-related physical or psychological changes result in specific risks for older workers [17]. Given that work behavior is one of the main causes of occupational accidents, an investigation into the effects of age-related characteristics on the safety behaviors of older construction workers is necessary. Therefore, this study aims to fill the gaps in the construction safety literature by investigating the factors and mechanisms underlying safety behaviors of older construction workers with considerations of their age-related characteristics. Potential results may be beneficial for construction managers and employers to enhance the safety behaviors of their older workers.

## 2. Literature Review

Safety behavior may be estimated better than safety outcomes as the latter often occur less frequently [18]. One stream of unsafe/safe behavior models has been introduced on the basis of the theory of individual performance and organizational climate. In such models [12,19], safety behavior is regarded as a type of individual performance. The precursors, such as safety climate, directly affect safety motivation and knowledge, which in turn influence safety performance. These models are grounded in the theory of performance of Campbell et al. [20], which identifies knowledge, skills, and motivation as the three proximal determinants of the performance of an individual. Researchers [21,22] have often combined knowledge and skill as a single term (i.e., safety knowledge) when delineating safety behavior. However, the performance theory-based safety behavior models have usually adopted a general or relatively rough measure of safety motivation, which may not encompass all aspects of motivation related to safety performance [21].

Theory of planned behavior (TPB), which is proposed by Ajzen [23] and extended from the Theory of Reasoned Action [24], is a rational decision-making theory that can explain numerous behaviors in specific contexts [10,25]. In contrast to the theory of performance, the TPB depicts intention/motivation through three dimensions of psychological drivers including attitude, subjective norms (SNs), and perceived behavioral control (PBC). Attitude is the degree to which a person exhibits a favorable evaluation of the behavior in question. SNs refer to the perceived social pressure to perform the behavior, whereas PBC refers to the perceived ease to perform the behavior of interest [23]. PBC is a generalized concept of competence belief, which contains but is not limited to the dimension of knowledge. Moreover, these psychological drivers are determined by background factors (e.g., individual, social, or organizational) [26]. In the revised TPB [26], the actual control factors are included and assumed to exhibit direct influences on PBC and behavior. The TPB has been applied in predicting unsafe behaviors of maintenance personnel in military services and safety behaviors of transportation workers [10,27]. Compared with a performance theory-based model, a TPB-based safety behavior model contains comprehensive and sophisticated dimensions of psychological drivers and depicts behaviors as a sociocognitive process. The lack of study on construction safety has utilized the TPB in modeling safety behavior.

## 3. Hypotheses and Theoretical Model: Applying the TPB in Modelling Safety Behaviors of Older Construction Workers

This study adopts the TPB as a theoretical framework to depict the safety behaviors among older construction workers. Safety behavior is classified into safety participation (SP) and safety compliance (SC) [12,19]. SP refers to the safety behaviors that are “frequently voluntary”, whereas SC refers to those that are “generally mandated” [21]. Psychological drivers of attitude, SNs, and PBC play the role as proximal determinants in modeling safety behavior based on the TPB [23]. In this study, the background factors or, alternatively, distal antecedents consist of organizational and personal factors as suggested by Christian et al. [28].

Management commitment (MC) and work pressure (WP) are selected as the primary organizational factors. Zohar found that a worker’s perception of his or her manager’s attitude toward safety is the most important determinant of safety climate [29]. Subsequently, the effects of MC on safety behavior have been explored, and their importance has been recognized [12,28,30]. Guo et al. indicated that when managers are believed to exhibit a high commitment to safety, their subordinates may want to meet management expectations by increasing their efforts in daily safety practices [12]. These beliefs are then socially transmitted to become collective norms and values within an organization [12]. WP can be regarded as a type of actual control factors. Mullen stated that workers behave unsafely partly due to the WP imposed by their supervisors [31]. Guo et al. argued that negative effects of conflicts exist between WP and safety [12]. To meet the work pace, workers may take shortcuts. Their WP may then lead to safety behavior violations.

The personal factors included in the model are safety knowledge (SK), aging expectation (AE), and health conditions (HCs). SK was found to be closely associated with safety behavior [12,21]. A meta-analysis study by Christian et al. indicated that SK poses a considerable positive synthesized effect on safety behavior [28]. HCs and AE are age-related physical and psychological characteristics of older construction workers, which may affect their behaviors. Literature has indicated that aging self-perceptions and multiple preventive health behavior are positively correlated even after the adjustment of important covariates [32]. The stereotype embodiment theory of Levy suggests that the aging process is socialized with strong biomedical influences [33]. This process has generated negative age stereotypes related to weakness, disorder, and disease. As adults age, they adjust their behaviors either consciously or unconsciously to match these stereotyped expectations in self-fulfilling ways [33]. Therefore, speculating whether the expectations of older construction workers on aging exhibit impacts on their safety behaviors is reasonable. Poor HCs of workers can increase accident risks at work [34]. Therefore, the influences of HCs on the safety behaviors of workers must be investigated as the latter is a critical cause of occupational accidents.

**Figure 1:**
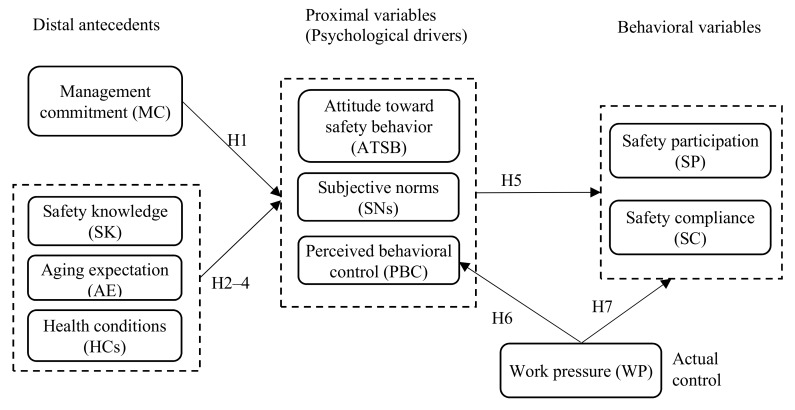
Older-construction-worker safety behavior model.

On the basis of the discussions above, the following hypotheses are proposed, and an old-construction-worker safety behavior model (OSBM) is developed for testing. Figure 1 illustrates the proposed OBSM.

**Hypotheses 1** **(H1).** *MC generates indirect effects on the safety behaviors of older construction workers via psychological drivers (i.e., attitude, SNs, and PBC)*.

**Hypotheses 2** **(H2).** *The SK of older construction workers affects their safety behaviors via psychological drivers*.

**Hypotheses 3–4** **(H3–4).** *The AE and HCs of older construction workers influence their safety behaviors via psychological drivers*.

**Hypotheses 5** **(H5).** *The psychological drivers of older construction workers mediate the effects of organizational/personal variables on safety behaviors, i.e., psychological drivers have direct effects on safety behaviors*.

**Hypotheses 6–7** **(H6–7).** *WP directly affects the PBC of workers and their safety behaviors*.

## 4. Methodology

### 4.1. Development of the Instrument

Ten subscales were adopted to measure the constructs involved in the theoretical model to test our hypotheses. The measurements of the HCs of the participants were developed with reference to McDowell [35], which had been validated by Chen and Chan [36]. AE measurements were extracted from the 12-item Expectations Regarding Ageing Survey (ERA-12) [37]. Four items among these covering three dimensions (physical health, mental health, and cognitive function) with highest factor loadings were chosen. The other eight construct items were developed by generating an item pool from the literature. HCs and AE items were not included in the pool due to the lack of existing study on safety behavior that examines age-related characteristics, leading to the limited context-suitable sources for these two constructs. Subsequent scientific item reduction procedures were conducted following those in Seo et al. [38].

#### 4.1.1. Development of an Item Pool

On the basis of the literature review, a 124-item pool was generated (Table 1). Positively and negatively worded items were likewise included as suggested by Pedhazur and Schmelkin [39]. These items had been screened for redundancy and clarity.

#### 4.1.2. Item Reduction

Through a content validity survey, the item reduction procedure was conducted by collecting the ratings on the quality of items for the corresponding instruments from five experts. These five experts included one senior researcher who has researched on construction workers and their risk-taking behaviors, three senior researchers who specialize in occupational safety and health, and one regional construction manager. Table 2 depicts an abbreviated content validity rating form. The definitions of the eight constructs were outlined. If a concept is the product of several domains/categories, then its multiple dimensions were included and defined. The items of a specific construct were randomly listed within the block of the same construct on the basis of the recommendation of Slocumb and Cole [45]. The five validators were requested to rate the content validity of each item in measuring the corresponding construct with respect to “relevance to the construct” [46] and “variability of the item in response” (i.e., all participants will not react with identical responses) [45] by using a 1–10 rating scale. The rating scores of relevance and variability given by the five experts were summed up for each item, with a range of possible results from 5 to 50. A qualified item was determined when both relevance and variability scores were greater than 30 (average on 6). The top five qualified items with highest relevance scores were selected. Only the qualified one was included if there were less than five qualified items. The sorted items were then carefully checked and compared to avoid redundancy in dimension. Table 3 shows the result details.

#### 4.1.3. Selection of Measurement Format

All the items were measured using the seven-point Likert scales. All items exhibited verbal anchors of “strongly disagree” and “strongly agree” at points 1 and 7, respectively, except those measuring the HCs. HCs items showed verbal anchors of “extremely bad” to “extremely good”. These measurements were used by Brown et al. [47], Ma et al. [48], and Seo et al. [38].

### 4.2. Demographic Information

The demographic information included in the questionnaire were age, gender, marital status, number of family members, education level, work skill level, work experience, and work status.

### 4.3. Sample Size and Data Collection

Structural equation modeling (SEM), which allows the simultaneous examination of a series of dependence relationships [51], was utilized to test the theoretical model of this study. Bollen recommended a sample of 150 or more for covariance-based SEM [52]. Marsh and Bailey indicated that the ratio of indicators to latent variables is a substantially better basis for calculating the sample size of SEM than merely obtaining the number of indicators [53], which is suggested by the criterion of 5–10 participants per indicator. Westland proposed a function of the ratio of indicator variables to latent variables in calculating the lower bounds on sample size in the SEM [54]. The minimum sample size for the indicator/latent ratio of the current study (i.e., 38/10) was 112, while an actual sample size of 260 was obtained. The involved questionnaires contained less than 5% missing responses and had unsystematic response patterns. The target participants were construction workers aged 50 years old or over, following the studies of Dong et al. [55] and Peng and Chan [3]. The questionnaires were collected with the assistance of local labor unions. Each participant received a HK$30 supermarket voucher upon completing the survey. All the respondents provided their informed consent before participating in the study. This research was approved by the Internal Review Board (IRB) of the City University of Hong Kong (approval number: 11,204,619).

### 4.4. Data Analyses

The missing values were filled in with the medium value related to each item. Data were analyzed through confirmatory factor analysis (CFA) and SEM using SPSS 24.0 and AMOS 24.0. Five goodness-of-fit indices, which contain incremental and absolute indices, were adopted to evaluate the fitness of measurement and structural models. These indices were the chi-square to its degree of freedom (χ^2^/df), Tucker–Lewis Index (TLI), comparative fit index (CFI), standardized root mean squared residual (SRMR), and root mean square error of approximation (RMSEA), as suggested by Hu and Bentler [56]. A χ^2^/df less than 3 suggests a good fit of the model [57]. The suggested criteria for TLI and CFI were higher than 0.9 [11]. The RMSEA and SRMR values of less than 0.08 indicate a reasonable fitness of the model [58]. Convergent validity was analyzed through the composite reliability and factor loading indicators. Discriminant validity was verified if the square root of average variance extracted (AVE) for a factor was greater than its largest inter-construct correlations [51].

## 5. Results 

### 5.1. Demographics

Table 4 shows the demographic profile of the 260 respondents. The average age and construction work experience of participants were 57.1 (±5.7) and 28.8 (±12.2) years, respectively. Most of them were male (95.8%). The largest proportion of the respondents were between 50 and 54 years old (36.5%), married (86.2%), skilled (86.8%), working full time (92.5%), had lower secondary education level (45.9%), and had four or more family members (55.4%).

### 5.2. Testing the Measurement Model

The results of CFA showed an excellent fit of the measurement model to the data (χ^2^ = 1106.9, *p* < 0.001, χ^2^/df = 1.798, TLI = 0.922, CFI = 0.931, RMSEA = 0.055, and SRMR = 0.051). Table 5 shows the convergent validity indices of the latent factors. All composite reliability values were higher than 0.7. All the factor loading estimates and AVE values were 0.5 or higher. Results indicated that the internal consistency of the measurement model was acceptable [51]. Table 6 reveals that the square root of AVE for a factor is greater than the largest correlation between the construct and another construct, thereby confirming the discriminant validity of the factors.

### 5.3. Testing the Structural Model

**Figure 2:**
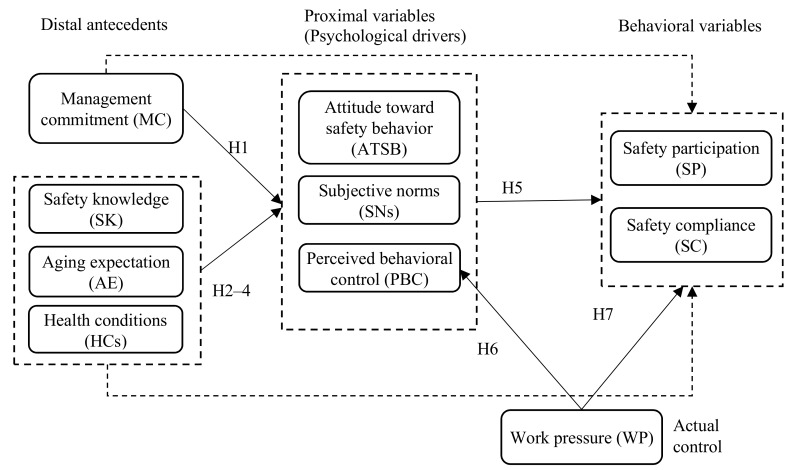
Conceptual Models 1 and 2 of older-construction-worker safety behaviors. Note: Direct effects (dotted arrow lines) of MC, SK, AE, and HCs on safety behavior were included in Model 1 but excluded in Model 2.

Two versions of the structural model were analyzed to test the mediating effects of psychological drivers between organizational/personal variables and safety behavior (Figure 2). In the first model (Model 1), the direct and indirect (through psychological drivers) effects of organizational and personal variables on safety behavior were hypothesized (i.e., partial mediation model). In the second model (Model 2), the direct effects were constrained to zero to allow psychological drivers to fully mediate the effects of distal antecedents on safety behavior (i.e., total mediation model). Given that Model 2 is nested within Model 1, they were compared by means of Δχ^2^ (Δdf) statistically to determine the final model to retain. The considerably parsimonious model (i.e., Model 2) should not be rejected if the Δχ^2^ (Δdf) is not significant at *α* of 0.01 [59].

The goodness-of-fit indices demonstrated that Models 1 and 2 exhibit good fitness to our data (Table 7). The path analysis results of Model 1 indicated that all the direct relationships between organizational/personal factors and safety behavior were insignificant aside from the relationship between MC and SC. Given that the full mediation model (i.e., Model 2) was more parsimonious and was not worse than the partial mediation model (Δχ^2^ (8) = 16.457, *p* = 0.036), Model 2 was retained as the final substantive model.

Table 8 depicts the direct, indirect, and total effects of related factors on safety behavioral variables. The explained variances for SP and SC in Model 2 were 74.2% and 63.1%, respectively. Among the distal antecedents, SK yielded the greatest total effects on SP and SC. However, the effects of HCs and WP on safety behavior were relatively insignificant.

**Figure 3:**
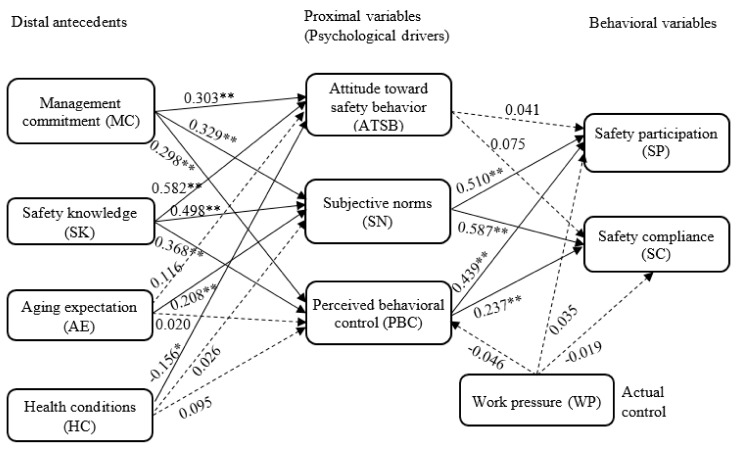
Finalized older-construction-worker safety behavior model. Note: Solid and dotted arrow lines represent significant and insignificant relationships, respectively; *: *p* ≤ 0.05; **: *p* ≤ 0.01.

Figure 3 shows the results of the Model 2 hypotheses. The solid arrow lines indicate significant relationships that passed the hypotheses tests. The details are described in the following paragraphs.

Hypothesis 1 proposed that MC poses effects on the psychological drivers of older construction workers, which was supported by the findings of this study. Figure 3 depicts that MC exhibited significantly positive influences on all the three psychological variables. Among these influences, the relationship between MC and SNs was the greatest.

Hypothesis 2 suggested that the SK of older construction workers influences their psychological drivers, which was also supported by the testing results. Findings revealed that SK generated significantly positive effects on all the three psychological drivers, while the effect on ATSB was the greatest.

Hypotheses 3–4 indicated the effects of the AE and HCs of workers on their psychological drivers. Both two hypotheses were partially supported. AE was found to be positively related with the SNs of workers, while its effects on other two psychological drivers were insignificant. HCs exhibited a significantly negative influence on the ATSB of workers, but it had no significant influence on the SNs or PBC of workers.

Hypothesis 5 proposed the mediating role of psychological drivers between organizational/personal and safety behavioral variables. The effects of ATSB on safety behavior were insignificant, whereas SNs and PBC were likewise positively related with safety behavior.

Hypotheses 6–7 proposed that WP generates direct effects on PBC and safety behavior. These two hypotheses were not supported by our findings. Findings revealed that WP has no significant influence on PBC or on safety behavior.

## 6. Discussion

This study primarily aimed to ascertain the factors and mechanisms underlying the safety behaviors of older construction workers. A TPB-based integrative conceptual model was established to explain the process through which organizational and personal factors influence the safety behaviors of older construction workers. In general, the conceptual model provided a considerably good explanation of the safety behaviors of older construction workers. Research hypotheses were fairly validated.

### 6.1. Mediation Role of Psychological Drivers and Their Impacts

The testing results of the two model versions indicated the equivalent fitness between the total (Model 2) and partial (Model 1) mediation models. Path analysis results showed that most direct relationships between organizational/personal and safety behavioral variables were insignificant. However, the relationships between organizational/personal variables and psychological drivers and between psychological drivers and safety behavior were both significant. Therefore, the total mediation model was supported. This finding implies that psychological drivers totally mediated the relationships between organizational/personal variables and safety behavior.

Mediation explains “how” and “why” an effect occurs [60]. In the present study, the effects and mechanisms of the three psychological drivers on influencing safety behavior were different. The attitudes of older construction workers showed insignificant influence on SP and SC. Ajzen indicated that attitude normally exhibits weak correlations with behavior given that any specific behavior reflects not only the influence of a relevant general disposition but also the factors unique to the particular situation and action being observed [23]. Iacuone claimed that a particular variety of hegemonic masculinity (e.g., real men are tough guys) exists in the building industry, which affects the relationships between workers hierarchically and their perceptions toward safety risks at work [61]. The prevailing ideology dictates that men should be willing to involve in dangerous activities [61]. Consequently, even the older construction workers with relatively positive ATSB (5.9/7 in this survey) may not act based on what they think is right. By contrast, SNs played a considerably positive role in promoting SP and SC for older construction workers. That is, those groups who value the importance of safety behavior can relatively resist the hegemonic masculinity existing in the construction industry and help their older workers to act safely. The positive roles of co-workers’ descriptive norms and supervisors’ injunctive safety norms to improve the safety behaviors of workers have also been confirmed by Fugas et al. [10]. Low et al. compared the working experience of construction workers between the accident and super-safe groups [15]. They found that workers of the accident and super-safe groups respectively attributed their unsafe and safe practices to the SNs of their co-workers. The PBC showed significantly positive effects on SP and SC. When workers believe that they have the competence to work safely, they tend to behave safely [10]. The current research indicates that the influences of PBC on SP were greater than those of SC. This finding is consistent with our anticipation. Fugas et al. suggested that SC ensures the control and enforcement of the rules, whereas SP facilitates using the discretion of workers concerning the safety of their work behaviors [10]. The latter is a higher level of the requirement of safety behavior than the former, which may be more related to informative influence.

### 6.2. Organizational and Personal Factors Affecting the Safety Behaviors of Older Construction Workers

#### 6.2.1. Management Commitment

Generally, a high management level exhibits a high impact on employee behavior. However, the relationship between the senior and middle management and employee behavior is neither direct nor unconditional [62]. Some researchers have shown that employees mainly comply with the instructions from the upper management [11,63]. Findings in this study affirmed previous statements and revealed that MC was positively correlated with all the three psychological drivers. That is, the higher the level of management commitment perceived by older construction workers, the better the attitude, the greater the SNs and the higher the level of PBC toward safety behavior they had, which in turn promote their safety behaviors.

#### 6.2.2. Safety Knowledge

Literature has suggested that SK is a significant factor that affects the safety behaviors of workers [12,28,41]. In this study, the standardized total effects of SK on safety behavior were of considerable magnitude (0.439 and 0.423 for SP and SC, respectively; see Table 8). Specifically, SK showed positive influences on all the three psychological drivers, which indicates that increasing SK would result in the improvement of the inner motivation and controllability of workers toward safety behavior. Guo et al. indicated that workplace safety depends upon the adaptive behaviors of workers given that construction jobs are highly dynamic and workers have a high degree of freedom to perform their tasks [12]. Szóstak also stated that the most important element of work safety in construction industry is worker [64]. A worker in the accident process plays the roles of decision maker, perpetrator and victim [64]. Providing adequate SK and skills are essential for workers to make proper decisions in avoiding accidents. Tacit SK obtained from work experience and injury exposure can significantly improve the hazard detection for construction workers [65].

#### 6.2.3. Aging Expectation and Health Conditions

Our results showed that negative AE was positively correlated with older construction workers’ SNs. Thus, older construction workers who have negative beliefs regarding aging tended to perceive high pressure of safety concerns from important others such as their families. Levy [33] and Levy and Myers [32] indicated that older people with negative age stereotypes tend to practice less preventive health behaviors because they perceive that deteriorated health problems are inevitable with aging; thus, healthy practices are futile. However, safety behavior seems to be conceptualized differently from health behavior by older construction workers in our study. Workers who have negative beliefs about aging might perceive that their important others would feel the same and thus perceive a high level of SNs, thereby promoting their safety behaviors.

Good HCs were found to be negatively correlated with the ATSB of older construction workers in the present study. The possible explanation is that those who have good HCs might have experienced few occupational accidents. The fortunate past experience might influence workers’ belief on risk-taking behavior by perceiving unsafe behavior as not that risky. Consequently, their attitude toward safety behavior became relatively undesirable.

#### 6.2.4. Work Pressure

Our WP results showed insignificant influences on the PBC and safety behavior of older construction workers. Consensus regarding the effects of WP on safety behavior was lacking among existing studies. For instance, Fogarty and Shaw indicated no direct link from workplace pressure to the violations of safety behavior for aircraft maintenance workers, and the indirect link between WP and violations was rather weak [27]. However, Guo et al. found that production pressure predicted SP and SC directly for general construction workers [12]. In the context of this study, WP has relatively weak impacts on altering the behaviors of older construction workers, which may be caused by control beliefs over work increasing with age [66]. On the basis of their work experience, older people may have acquired more coping resources and may therefore appraise situations as less stressful and report fewer hassles than their young counterparts [67,68]. Therefore, inferring that older construction workers commonly handle their pressures well is reasonable; thus, the influences of WP on their behaviors are relatively weak or insignificant.

### 6.3. Implications and Limitations

#### 6.3.1. Theoretical Implications

Theoretically, this study filled the gaps in the construction safety literature by examining the safety behaviors among older construction workers. Our findings provided evidence of organizational and personal factors underlying the safety behaviors of older construction workers with considerations of their age-related characteristics. A TPB-based OSBM was developed and validated. The integrative model showed considerable explanatory accounts of safety behaviors among older construction workers within a social–cognitive rational action framework. This model facilitates an understanding of psychosocial drivers that explain “how” and “why” such behaviors occur.

#### 6.3.2. Practical Implications

Fugas et al. claimed that an understanding of psychosocial factors is beneficial in implementing an effective and successful safety management strategy [10]. Our findings demonstrated the critical roles of SNs and PBC on the safety behaviors of older construction workers. Antecedents that could influence the SNs and PBC of workers should be highly valued. For instance, the safety concerns from the family members or close friends of older construction workers would be helpful in promoting their SNs. The safety culture or atmosphere shared within the work team might also be an important determinant of SNs and could resist the masculinity prevailed in the construction industry. In addition, negative AE can protect older construction workers from performing risk-taking behaviors to some extent by influencing their SNs. PBC also showed considerable impacts on the safety behaviors of older construction workers. Therefore, improving the competence of workers can facilitate their mandatory and voluntary safety behaviors. MC and SK were the two critical distal antecedents that influence all three psychological drivers and should be highly recognized. Improving MC requires the management to value safety and to engage in communication and actions that support safety. Providing resources and setting policies to make safety a priority would be effective ways to promote safety. Regarding SK, Burke et al. indicated that a sound safety training program is effective in improving the SK of employees [69]. Vinodkumar and Bhasi found that training, communication, feedback, and rules and procedures for safety were all predictors of SK [22]. Mohammadfam et al. argued that supervisors can aid the familiarization of novice workers with the hazards at their work [41].

#### 6.3.3. Limitations

The limitations of this study were acknowledged. First, although the TPB-based conceptual model showed good explanation of the safety behaviors of older construction workers, the investigated organizational and personal factors may not be a comprehensive cover of related determinants. In particular, the involved age-related characteristics (AE and HCs) were found to exhibit moderate or weak associations with the safety behaviors of older construction workers. Therefore, a further examination of other age-related characteristics that influence the safety behaviors of older construction workers is necessary. Second, previous research has indicated that organizational-level factors influence group- and personal-level factors [12]. However, the interrelationships between organizational and personal factors were not considered in this study. For a cross-sectional survey, we only considered the current levels of organizational and personal factors reported by respondents and the associations with psychological drivers and safety behaviors.

## 7. Conclusions

This study examined the factors and mechanisms underlying the safety behaviors of older construction workers. The influences of organizational and personal factors, particularly age-related characteristics on the safety behaviors of older construction workers, were quantified. The results highlighted the critical roles of MC, SK, and AE on the impact of safety behaviors for older construction workers. In addition, a TPB-based OSBM was established. The conceptual model confirmed the mediating role of the psychological drivers and showed considerable explanatory accounts for the safety behaviors of older construction workers within a social–cognitive rational action framework. This model facilitated an understanding of mechanisms underlying these behaviors. On the basis of our findings, new insights into the safety behaviors of older construction workers can be provided. Findings will help construction management to develop work improvements and interventions in reducing occupational accidents among older workers.

## Figures and Tables

**Table 1:** Sources of item pool.

Subscales	Sources
(1) Management commitment	[12,13,22,30,40,41]
(2) Work pressure	[12,41,42]
(3) Safety knowledge	[12,22,41]
(4) Attitude towards safety behavior	[10,13,41]
(5) Subjective norms	[13,43]
(6) Perceived behavior control	[10,13,44]
(7) Safety participation	[10,12,13,22,41]
(8) Safety compliance	[10,12,22,30,40]

**Table 2:** Abbreviated content validity rating form.

Instructions		
You will find the list of 124 items extracted from existing studies on unsafe/safe behaviors below. These items will be used to measure eight constructs, including (1) management commitment, (2) work pressure, ……, (8) safety compliance.		
Please familiarize yourself with the constructs and their definitions first. Thereafter, read each item carefully and rate its content validity in measuring the corresponding construct in terms of “relevance to the construct” and “variability of the item in response”. Please indicate your answer on a 1–10 scale, with “1” indicating the lowest level and “10” indicating the highest.		
(Construct 1) Management commitment: the extent to which employees perceive that management values safety and engages in communication and actions that support safety.	Part I Relevance to the construct	Part II Variability of the item in response
Item 1a. Management allocates enough resources (time and effort) to safety.		
Item 1b. Following safe work practice is appreciated by the management.		
Item 1c. ……		

**Table 3:** Definitions and sorted items for constructs.

Constructs	Definitions (and/or Dimensions) of Constructs	Items
Management commitment (MC)	The extent to which employees perceive that management values safety and engages in communication and actions that support safety [28].	Corrective action is taken when the management is told about unsafe practices [22]. Management is concerned about our well-being [49]. Following safe work practice is appreciated by the management [41]. Management allocates enough resources (time and effort) to safety [13]. Management encourages employees here to work in accordance with safety rules despite the tight work schedule [50].
Work pressure (WP)	The extent to which work pressure overwhelms the ability of an individual to perform safely [41,42].	Shortcuts and risk taking are common due to heavy workload [42]. Doing a job while following all the safety rules is difficult [42]. We are often in such a hurry that safety is temporarily overlooked [42]. Time pressure is one of the reasons why employees tend to behave unsafely [41].
Safety knowledge (SK)	The extent of equipping requisite knowledge in terms of safety rules and procedures; use of safety equipment; identification of related hazards; and concepts of unsafe behaviors, conditions, and accidents.	I know how to use safety equipment and standard work procedures [22]. I know the hazards associated with my jobs and the necessary precautions to be taken while doing my job [22]. I have good knowledge about the concept of unsafe behavior, unsafe condition, near miss, and minor accidents [41].
Aging expectation (AE)	Expectations regarding aging in terms of physical health, mental health, and cognitive functioning [37].	The human body is like a car: When it gets old, it wears out [37]. As people age every year, their energy levels slightly decrease [37]. As people get older, they worry more [37]. Forgetfulness is a natural occurrence when growing old [37].
Health conditions (HCs)	This concept is measured with respect to five aspects, including general health status, health conditions compared with the same-age groups, physical work capacity, physical work capacity compared with the same-age groups, and psychological status.	How are your general health conditions [35]? How are your health conditions compared with the same-age groups? How do you rate your current work ability with respect to the physical demands of your work? How is your physical work ability compared with the same-age groups? How do you rate your current psychological status?
Attitude toward safety behaviors (ATSB)	The degree to which a person has a favorable evaluation of safety behavior [23].	In my job, compliance with safety rules is beneficial [10]. In my job, actively participating in safety rules is relevant [10].
Subjective norms (SNs)	Subjective norms refer to the perceived social pressure to perform safety behavior [23].	My family members and friends who are important to me would encourage me to work safely [13]. My colleagues whose opinion I value would approve my safe work behavior [13]. My team demonstrates to each workforce that they value their contribution to health and safety [13]. I prefer to work safely because people who are important to me would like me to do so. (Newly created, with the reference of Pender and Pender [43].)
Perceived behavioral control (PBC)	The perceptions of respondents of the extent to which they are capable of performing safety behaviors [23].	For me, working safely is easy [10]. I feel that I do not have control over the safety performance on my job [10]. I can successfully control over the working conditions (resources, facilities, and working area) that enable me to work safely [13]. I can successfully control over the work processes within my workplace [13].
Safety participation (SP)	Safety participation involves helping coworkers, promoting workplace safety programs, demonstrating initiative, and putting effort into improving workplace safety [19].	I encourage my co-workers to work safely [22]. I voluntarily carry out tasks or activities that help improve workplace safety [22]. I immediately report hazards or any incidences whenever I found one at work [13]. When I have a suggestion for modifying unsafe conditions, I share it with the safety department [41].
Safety compliance (SC)	Safety compliance involves adhering to safety procedures and completing work in a safe manner [19].	I follow correct safety rules and procedures while carrying out my job [22]. I use the appropriate personal protective equipment (PPE) as indicated by the Department of Safety and Health [10]. I properly perform my work while wearing PPE [10].

**Table 4:** Respondents’ demographic profile (N = 260).

Categories	Mean/Frequency	Percentage (%)	No. of Valid Values
**Work experience**	28.8 ± 12.2 years		246
**Age**	57.1 ± 5.7 years		260
(1) 50–54 years	95	36.5	
(2) 55–59 years	83	31.9	
(3) 60–64 years	51	19.6	
(4) 65–69 years	20	7.7	
(5) 70+ years	11	4.2	
**Gender**			259
(1) Male	248	95.8	
(2) Female	11	4.2	
**Education level**			255
(1) Preprimary	9	3.5	
(2) primary	50	19.6	
(3) Lower secondary	117	45.9	
(4) Higher secondary	62	24.3	
(5) Postsecondary	17	6.7	
**Marital status**			254
(1) Unmarried	20	7.9	
(2) Married	219	86.2	
(3) Divorced/Separated/Widowed	15	5.9	
**Skill**			242
(1) Semi-skilled	32	13.2	
(2) Skilled	210	86.8	
**Work status**			252
(1) Full time	233	92.5	
(2) Part time	19	7.5	
**No. of Family members**			249
(1) One member (live alone)	16	6.4	
(2) Two members	34	13.7	
(3) Three members	61	24.5	
(4) Four members or more	138	55.4	

**Table 5:** Convergent validity indices of the measurement model.

Construct	Item	Factor Loading	Composite Reliability	Average Variance Extracted (AVE)
**Management commitment (MC)**	MC1	0.839	0.922	0.704
	MC2	0.862		
	MC3	0.785		
	MC4	0.865		
	MC5	0.843		
**Work pressure** **(WP)**	WP1	0.742	0.850	0.587
	WP2	0.782		
	WP3	0.843		
	WP4	0.689		
**Safety knowledge (SK)**	SK1	0.866	0.840	0.641
	SK2	0.875		
	SK3	0.638		
**Aging expectation (AE)**	AE1	0.838	0.821	0.546
	AE2	0.921		
	AE3	0.573		
	AE4	0.554		
**Health conditions (HCs)**	HC1	0.836	0.886	0.611
	HC2	0.866		
	HC3	0.807		
	HC4	0.670		
	HC5	0.710		
**Attitude toward safety behaviors (ATSB)**	ATSB1	0.932	0.891	0.804
	ATSB2	0.860		
**Subjective norms (SNs)**	SN1	0.852	0.925	0.756
	SN2	0.867		
	SN3	0.853		
	SN4	0.905		
**Perceived behavioral control (PBC)**	PBC1	0.627	0.816	0.529
	PBC2	0.748		
	PBC3	0.861		
	PBC4	0.650		
**Safety participation (SP)**	SP1	0.844	0.877	0.641
	SP2	0.798		
	SP3	0.725		
	SP4	0.830		
**Safety compliance (SC)**	SC1	0.751	0.909	0.771
	SC2	0.924		
	SC3	0.946		

**Table 6:** Inter-factor confirmatory correlations among latent variables.

	MC	WP	SK	AE	HCs	ATSB	SNs	PBC	SP	SC
MC	0.839									
WP	−0.15 *	0.766								
SK	0.471 **	0.024	0.801							
AE	0.237 **	0.256 **	0.631 **	0.739						
HCs	0.463 **	0.077	0.609 **	0.272 **	0.782					
ATSB	0.521 **	0.021	0.661 **	0.503 **	0.373 **	0.897				
SNs	0.602 **	0.025	0.749 **	0.601 **	0.535 **	0.791 **	0.869			
PBC	0.508 **	−0.066	0.541 **	0.326 **	0.446 **	0.450 **	0.616 **	0.727		
SP	0.580 **	0.018	0.720 **	0.511 **	0.521 **	0.612 **	0.770 **	0.727 **	0.801	
SC	0.591 **	−0.021	0.623 **	0.465 **	0.511 **	0.619 **	0.757 **	0.583 **	0.798 **	0.878

Note: *: *p* ≤ 0.05; **: *p* ≤ 0.01; the diagonal value refers to the square root of AVE of the construct.

**Table 7:** Summary of goodness-of-fit indices for Models 1 and 2.

	χ^2^	Df	χ^2^/df	*p*-Value	TLI	CFI	RMSEA	SRMR
Model 1	1192.94	626	1.906	<0.001	0.910	0.919	0.059	0.056
Model 2	1209.40	634	1.908	<0.001	0.909	0.918	0.059	0.057
Model comparison	Δχ^2^ (8) = 16.457, *p* = 0.036							

**Table 8:** Direct, indirect, and total effects of related factors on safety behavioral variables.

	Effect Type	HCs	MC	AE	SK	WP	PBC	SNs	ATSB
SP	Direct effect					0.035	0.439	0.510	0.041
	Indirect effect	0.049	0.311	0.120	0.439	−0.020			
	Total effect	0.049	0.311	0.120	0.439	0.015	0.439	0.510	0.041
SC	Direct effect					−0.019	0.237	0.587	0.075
	Indirect effect	0.026	0.287	0.136	0.423	−0.011			
	Total effect	0.026	0.287	0.136	0.423	−0.030	0.237	0.587	0.075
